# Misconceptions, prejudice and social distance towards people with alcohol use disorders in China

**DOI:** 10.1186/s12888-025-06595-9

**Published:** 2025-02-19

**Authors:** Qingyan Yang, Nicolas Rüsch, Kebing Yang, Yajuan Niu, Yanxia Xiao, Yanfang Zhou, Yunlong Tan, Yi Zhang, Patrick W. Corrigan, Ziyan Xu

**Affiliations:** 1 Peking University Huilongguan Clinical Medical School, Beijing Huilongguan Hospital, Beijing, China; 2 Department of Psychiatry and Psychotherapy II, University of Ulm and BKH Günzburg, Ulm, Germany; 3 Tianjing Binhai New District Anding Hospital, Tianjin, China; 4 Department of Psychology, Illinois Institute of Technology, Chicago, USA; 5 Department of Psychiatry and Psychotherapy III, University of Ulm, Leimgrubenweg 12-14, 89075 Ulm, Germany

**Keywords:** Alcohol use disorders, Public stigma, Stereotype, Discrimination, China, Stigma change

## Abstract

**Purpose:**

Research from Western societies indicates pervasive public stigma against people with alcohol use disorders (AUDs). However, there is a lack of knowledge about the interactions between different components of stigma and their contribution to discriminatory behaviour towards individuals with AUDs within the Chinese cultural context. The aim of the present study was therefore to investigate the relationships of (mis-)conceptions, stereotypes, emotional reactions, perceived public stigma and their contribution to the desire for social distance among the Chinese adults.

**Methods:**

(Mis-)conceptions, stereotypes, emotional responses, willingness to help and avoid, perceived stigma, and the desire for social distance from individuals with AUDs were assessed via a cross-sectional online survey with a sample of 1,100 adults from the Chinese population.

**Results:**

Three-fourth of the participants recognised AUDs as mental illnesses, though 70% of the sample did not support health insurance coverage for the treatment of AUDs. A stronger desire for social distance was associated with greater recognition of alcohol use disorders as mental illnesses, heightened perceptions of dangerousness and personal responsibility, increased fear, and more willingness to avoid and withhold help, controlling for age, gender, education, employment, mental health service utilisation, and alcohol consumption. Fear fully mediated the effects of perceptions of dangerousness and responsibility on the desire for social distance.

**Conclusions:**

To improve social integration for individuals with AUDs in China, culturally tailored anti-stigma programmes are needed to reduce negative attitudes and discriminatory behaviours among the Chinese population.

## Introduction

Alcohol use disorders (AUDs) are one of the most prevalent mental disorders globally and are associated with high mortality and burden of diseases [1]. In China, the lifetime and 12-month prevalence of any alcohol use disorder was 4.4% and 1.8%, respectively, which was more than that of any drug use disorders [2]. However, many individuals with symptoms consistent with AUDs remain undiagnosed and untreated worldwide [3], including in China, where only 2.4% of individuals with alcohol dependence receive treatment [4]. Recent studies [5–7] indicate that alcohol-related stigma might be one of the most prominent barriers to treatment-seeking for alcohol use disorders.

Myths about AUDs are prevalent and can lead to misconception and stigma, further impeding the willingness of people with AUDs to seek the necessary help and support [8]. Common myths may involve the perception that alcohol use disorders result from personality weakness or character flaws, rather than recognising these disorders as mental illnesses influenced by a complex interplay of biological, psychological, and environmental factors [9]. Some people may believe alcohol-related treatment should not be covered by medical insurance [10]. These oversimplified perspectives can contribute to judgmental attitudes and discourage individuals from seeking help, exacerbating discrimination towards individuals with these conditions and hinder their recovery process.

Public stigma is markedly encountered by people with AUDs and often leads to additional challenges in their lives [11]. Stigma towards AUDs encompasses widely endorsed negative stereotypes, leading to prejudice and discrimination [12]. Common stereotypes about people with AUDs are that they lack self-control, are dangerous and are emotionally unstable [11, 13]. According to the attribution theory of mental illness stigma [14, 15], people who attribute person’s problems to controllable factors are more likely to be hostile and critical towards this individual. Compared to individuals with psychosis, people with AUDs are judged as much more responsible for their conditions and less worthy of support or care [13]. Prejudice occurs if people endorse these stereotypes and react emotionally. For example, “It is right that people with alcohol use disorders are unreliable and dangerous. I am scared of them” [12]. Discrimination involves unjustified unequal treatment or actions taken towards the stigmatised individuals or groups [16], such as limitations to rental opportunities, employment prospects and interpersonal relationships [17]. Studies among the general population [18, 19] suggested a connection between higher levels of stereotypes and a greater preference for social distance from individuals with AUDs.

Perceived stigma refers to the subjective perception of the stereotypes, prejudice and discrimination held by the majority population [20], which pertains to how stigmatised individuals are viewed and treated by society. It is deeply rooted in public stigma and reflects the anticipated stigmatising attitudes among the general public, where individuals fear being judged, blamed, or socially excluded if they were experiencing alcohol use disorders [21, 22]. Findings from a German national survey [23] suggested a potential association between perceived stigma and the tendency for social distancing from individuals with AUDs. Consequently, perceived stigma may discourage individuals from pursuing the necessary help for their alcohol use problems [24], contribute to social withdrawal to avoid judgement from others [25], and lead individuals to internalise stereotypes, thereby diminishing self-worth [26, 27]. Other types of stigma, such as structural stigma and self-stigma, are also significant factors that can impact individuals with AUDs [13], though these are beyond the scope of this study.

Although drinking alcohol is a widely accepted cultural tradition and crucial for fostering social bonding in China, traditional collectivist culture emphasises morality and self-controlled drinking, stressing maintenance of social order and the avoidance of embarrassment with drunken behaviours [28]. Furthermore, the concept of “saving face” is deeply rooted in Chinese culture [29]. Social harmony and the reputation of the family and community are highly valued. Individuals with AUDs may be viewed as bringing shame not only upon themselves but also upon their families [30]. Feeling ashamed, and fearing being labelled and discriminated against were identified as key factors contributing to reluctance to seek help and social withdrawal among individuals with substance use disorders [31]. These cultural norms, subject to stigma and social marginalization [32], are amplified by the collectivistic nature of Chinese society, where individuals tend to perceive public prejudices as justified [33]. Stigma towards AUDs may therefore be particularly widespread in China.

Although stigma surrounding mental illnesses like depression and schizophrenia has been extensively studies in China [34], stigma associated with AUDs presents unique challenges that remain inadequately addressed. While a few studies conducted among the Chinese population have provided initial insights into public stigma and its potential impacts on individuals with AUDs, they often lack a comprehensive and multidimensional perspectives. A community-based study [31] showed that the vast majority of respondents perceived individuals with alcohol addiction as having undesirable characteristics, which could potentially hinder their willingness to seek help. A cross-sectional study [35] highlighted an association between alcohol use problems and experience of unfair treatment in public services. Xiong et al. [36] found that the general public expected occupational restrictions of people with a history of alcohol use problems, particularly in roles requiring high responsibility, such as that of a bus driver.

These population-based surveys shed light on specific aspects of alcohol-related stigma, however, they failed to consider stigma as a multidimensional concept that encompasses various interconnected components, including stereotypes, prejudice, and discrimination [12, 13]. Furthermore, (mis-)conception may shape stereotypes and emotional reactions, particularly in cultural contexts like China, where misinformation about AUDs is widespread [30, 33]. Discriminatory behaviour represents a critical manifestation of stigma, significantly affecting individuals’ access to treatment, social integration, and overall well-being [17]. The interplay between different components of stigma and their contribution to discriminatory behaviour in the Chinese cultural context remain unclear. While emotional reactions have been found to mediate the relationship between stereotypes and discriminatory behaviours in studies examining common mental health problems among Chinese population [37, 38], whether this dynamic extends to individuals with AUDs has yet to be explored. Further research in this field is needed to inform targeted interventions that address alcohol-related stigma.

The present study therefore aimed to test the hypothesis that a higher level of desire for social distance from individuals with AUDs is independently associated with more (mis-)conceptions, perceived public stigma, stereotypes, and negative emotional reactions among Chinese adults. Building on Corrigan et al.’s attributional model of mental illness stigma [14], the study adapts this framework to the Chinese cultural context to investigate the mechanism underlying the desire for social distance. Specifically, we examined whether (mis-)conceptions, perceived public stigma, and stereotypes (e.g. perceived dangerousness and responsibility) contribute to avoidance and reduced willingness to help, which in turn lead to a higher level of desire for social distance from individuals with AUDs, both directly and indirectly through emotional reactions such as pity, anger, and fear. As female gender, older age, lower education, and unemployment were found to be associated with higher public stigma about mental illness among the Chinese population [39], we controlled age, gender, education, and employment to avoid confounding effects. Additionally, given the association of alcohol consumption and mental health utilisation with alcohol-related stigma [40], we also included these as confounding variables. Findings from the study could help guide the development of targeted interventions to reduce stigma and improve social integration for individuals with AUDs among the Chinese population.

## Methods

### Design and participants

This cross-sectional online survey employed a convenience sample of Chinese adults aged 18 years and older. A total of 1,124 participants initially submitted the survey via the Wenjuanxing platform, a professional online survey tool in China often compared to Amazon Mechanical Turk in terms of functionality and use. Recruitment was conducted through advertisements on social media platforms, dissemination via community newsletters, and a combination of convenience and snowball sampling methodologies. For the snowball sampling, individuals initially recruited through convenience methods were encouraged to share the study link with others who might meet the study criteria. All participants were subsequently invited to refer the survey to other individual within their personal or professional networks. This recruitment strategy aimed to ensure a diverse and voluntary participant pool without financial compensation.

### Measurements

The survey was conducted entirely in Chinese. We provided a description of “alcohol use disorders” in the informed consent to ensure participants had a clear understanding of the term, which was consistently used throughout the survey. To ensure clarity and cultural relevance, all survey items were pretested with a sample from the target population.

Adapted from Schomerus et al.’s study [19] on public views towards individuals with AUDs, we used the following empirical questions regarding (mis-)conceptions about AUDs: Whether they think alcohol use disorders are mental illnesses (0 = no, 1 = yes); Do they believe that alcohol use disorders are due to a lack of willpower (0 = no, 1 = yes); What do they think is the possible cause of an alcohol use disorder (1 = biogenetic, 2 = psychosocial, 3 = biopsychosocial, 4 = others); Whether they think the treatment for alcohol use disorders should be covered by health insurance (0 = no, 1 = yes).

The Attribution Questionnaire (AQ [14]), was used and adapted to measure stereotypes and emotional responses towards individuals with alcohol use problems. The AQ has been employed in the Chinese population to assess stigmatising attitudes towards individuals with schizophrenia [41] and children with autism [42]. Both adoptions demonstrated robust psychometric properties. In this study, the vignette in the original AQ was modified to depict David, a 32-year-old single male. He is a company employee and currently lives with his parents. Due to his alcohol use disorders, he had been hospitalised for treatment multiple times. After reading the vignette, participants were given eight questions on dangerousness, responsibility, treatment optimism, pity, anger, fear, willingness to help, and avoidance. Items were coded on a 9-point Likert scale from 1 = “none at all” to 9 = “very much” (Cronbach’s alpha = 0.73).

Perceived stigma was measured using a Chinese version of the Perceived Stigma towards People who use Substances [43], which is a cross-cultural validation of the 8-item Perceived Stigma of Addiction Scale [44]. Items were scored from 1 to 4 with higher scores representing stronger agreement with the statements. To specifically measure stigma associated with alcohol use disorders, the term “substance use concerns” was replaced by “alcohol use disorders” in this study (Cronbach’s alpha = 0.79).

The desire for social distance, as a proxy for discriminatory behaviour, was measured by the Link et al.’s Social Distance Scale [20]. This scale has been employed in Chinese samples and has shown good psychometric properties [45]. It comprises 7 items (e.g. “how willing are you to have someone like David as a neighbour? ) on a 4-point Likert scale ranging from 0 (definitely willing) to 3(definitely unwilling), with higher scores representing a greater desire to distance oneself from persons with AUDs (Cronbach’s alpha = 0.90).

Alcohol consumption was assessed using the brief version of the Alcohol Use Disorders Identification Test - Consumption (AUDIT-C [46]), which included the following three questions: (a) How often do you have a drink containing alcohol? (b) How many drinks did you have on a typical day when you were drinking in the past year? (c) How often did you have 6 or more drinks on one occasion in the past year? The AUDIT-C was scored on a scale ranging from 0 to 12, with higher scores indicating higher levels of alcohol consumption. Risky drinking was defined using an optimal cut-off of ≥ 5 for males and ≥ 4 for females in the Chinese population [47].

### Procedure

The study protocol was fully reviewed and approved by the Research & Ethics Committee of Peking University Clinical Medical School, Beijing Huilongguan Hospital, China. All prospective participants were informed about the study’s aims and provided online consent prior to participation. Anonymity and confidentiality were ensured throughout the study. The online survey was designed to take approximately 5 to 10 min to complete. Participants were required to complete all survey questions before submission, and incomplete submission were automatically excluded. Participants did not receive compensation for their involvement in the survey. To enhance the validity of this survey, several quality control measures were implemented. Submission completed in less than 2 min were excluded to account for insufficient response time. IP address verification was conducted to identify and exclude multiple submission from the same IP address. Additionally, reverse-coded questions were used to assess response consistency, and participants were excluded if they provided highly contradictory responses. For example, a participant who strongly agreed with the statement “Most people would willingly accept someone who has alcohol use disorders as a close friend” but also strongly agreed with the statement “Most people think less of a person who has alcohol use disorders” would be flagged for inconsistent responses. Twenty-four participants were excluded due to data quality concerns: 6 for insufficient completion time, 5 for duplicated submissions, and 13 for inconsistent responses to reverse-coded questions. Data were collected from March 1st to 15th, 2022.

### Analyses

First, we used descriptive statistics to analyse individual items, determining the extent to which answers to each item are related. Second, Spearman correlations and chi-square tests were used to analyse the correlations between misconceptions, stereotypes, emotional reactions, perceived stigma, and the desire for social distance. As the variable of the potential causes of AUDs contains multiple categories, we treated it as a nominal variable, employing either chi-square test or ANOVA for further correlation analyses. Third, we used multiple linear regression analysis to examine the independent associations of predictor variables with the desire for social distance as the dependent variable, controlling for age, gender, education, employment, alcohol consumption and lifetime mental health service utilisation. Fourth, to further explore the mechanisms underlying the associations with the desire for social distance, we conducted a path analysis using MPlus 8.3 with Maximum Likelihood Estimation. This analysis included only the variables that were found to be significantly and independently associated with the desire for social distance in the multiple linear regression analysis, testing both their direct and indirect effects. Specifically, we assessed whether emotional reactions mediated the relationship between misconceptions, perceived stigma, stereotypes, and the desire for social distance. Model fit was evaluated using the chi-square test (𝛘^2^/df < 2, *p* > 0.05), Comparative Fit Index (CFI > 0.95), Tucker–Lewis Index (TLI > 0.95), Standardized Root Mean Square Residual (SRMR < 0.08), and Root Mean Square Error of Approximation (RMSEA < 0.06) [48].

As the variable representing the potential causes of alcohol use disorders contains multiple categories, we used dummy independent variables for each non-reference category in the regression model. Multicollinearity was assessed using variance inflation factor (VIF) for each independent variable, with a VIF value 2.5 or above indicating multicollinearity [49]. The data were analysed using Statistical Program for Social Science (SPSS) - version 29. A two-tailed significance of *p* < 0.05 was used in the analyses. Specifically, for correlation analyses, a more stringent threshold of *p* < 0.01 was employed to bolster the reliability of the correlation results. A correlation coefficient of 0.10, 0.30, and 0.50 or higher indicates a small, moderate, or large effect size, respectively [50].

## Results

After data cleaning, the final sample consisted of 1,100 participants across 34 province-level administrative regions in China. The vast majority of the participants were from the Han Ethnic group. Nearly 60% of the participants were women. The socio-demographic characteristics of participants are presented in Table 1. In this study, 29.6% of males and 4.9% of females were identified as risky drinkers, which reflects an average level within the general population [51].

**Table 1:** Socio-demographic characteristics of participants (*N* = 1,100)

Variable	*N* (percentage)/M (SD)
Age	39.3 (10.1)
Gender, female	657 (59.7%)
Ethnic groups	
Han Manchu Korean Hui Mongolian Others	1053 (95.7%) 25 (2.2%) 5 (0.5%) 4 (0.4%) 4 (0.4%) 9 (0.8%)
Education (years)	
8–9 10–12 13–16 > 16	48 (4.4%) 66 (6.0%) 690 (62.7%) 296 (26.9%)
Married	826 (75.1%)
Unemployed	25 (2.3%)
Alcohol consumption (0–12)	2.3 (2.6)
Any mental health service use, lifetime	140 (12.7%)

Nearly three-quarters of participants recognised AUDs as mental illnesses, though approximately one-third held the belief that individuals with AUDs lacked willpower. Over 60% of participants endorsed a biopsychosocial model of alcohol use disorders. It is noteworthy that nearly 70% of the sample did not support health insurance coverage for alcohol-related treatment (Table 2).

**Table 2:** Descriptive statistics (*N* = 1,100)

Variable	*N* (Percentage)/ M ± SD (Range)
AUDs^a^ are mental illnesses (yes)	820 (74.5%)
AUDs are due to a lack of willpower (yes)	345 (31.4%)
Potential causes of AUDs	
Bio-psycho-social Psychosocial Biological Others	700 (63.6%) 235 (21.4%) 16 (1.5%) 149 (13.5%)
Supporting health insurance coverage (yes)	338 (30.7%)
Attributions^b^	
Dangerousness	7.28 ± 1.87 (1–9)
Responsibility	7.63 ± 1.66 (1–9)
Treatability	7.04 ± 1.98 (1–9)
Willingness to help	5.46 ± 2.36 (1–9)
Avoidance	5.35 ± 2.43 (1–9)
Pity	5.87 ± 2.75 (1–9)
Anger	5.84 ± 2.52 (1–9)
Fear	6.32 ± 2.28 (1–9)
Perceived stigma^b^	23.96 ± 4.39 (9–36)
Desire for social distance^b^	24.68 ± 3.69 (7–28)

^a^AUD = Alcohol use disorders; ^b^A higher score indicates a higher level of the measured variables

In bivariate relationships, the desire for social distance was markedly associated with a higher level of perceived stigma, moderately associated with more perceived dangerousness, avoidance and higher levels of fear and anger, while there is a weak correlation between the desire for social distance and less willingness to help. Increased perceived dangerousness was highly associated with more perceived responsibility and a higher level of fear. The recognition that AUDs are mental illnesses was slightly associated with a biopsychosocial attribution and greater perceived treatment optimism; however, it was mildly correlated with a greater endorsement of weakened willpower, higher levels of pity and fear, as well as increased perceived stigma and a desire for social distance. Individuals attributing the cause to biogenetic perceived the lowest level of anger, while those with biopsychosocial perspectives were more likely to support health insurance coverage for alcohol-related treatment. More withholding help was associated with higher levels of pity, anger and fear, avoidance, and perceived stigma, while more willingness to help was significantly associated with more perceived dangerousness and treatment optimism. Increased perceived stigma was associated with higher levels of anger and fear, respectively, but neither was associated with pity (Table 3).

**Table 3:** Correlations between misconceptions, stereotypes, emotional reactions, perceived stigma and the desire for social distance (* *p* < 0.01; ** *p* < 0.001)

		1	2	3	4	5	6	7	8	9	10	11	12	13
1	AUDs^a^ are mental illnesses													
2	Lack of willpower	0.20^**^												
3	Potential causes of AUDs^a, b^	0.12^**c^	-0.05											
4	Supporting health insurance coverage	0.04	-0.03	0.10^*c^										
5	Dangerousness	0.07	0.05	0.02	-0.02									
6	Responsibility	0.05	0.08^*^	-0.02	-0.10^*^	0.53^**^								
7	Treatability	0.18^**^	0.05	-0.02	0.001	0.45^**^	0.40^**^							
8	Willingness to Help	0.05	0.01	0.07	0.08^*^	0.17^**^	0.07	0.32^**^						
9	Avoidance	0.04	0.12^**^	-0.04	-0.12^**^	0.26^**^	0.27^**^	0.11^**^	-0.22^**^					
10	Pity	0.09^*^	0.02	0.04	0.04	0.32^**^	0.19^**^	0.26^**^	0.29^**^	0.03				
11	Anger	0.03	0.14^**^	0.09^*d^	-0.06	0.42^**^	0.40^**^	0.31^**^	0.29^**^	0.27^**^	0.29^**^			
12	Fear	0.07^*^	0.12^**^	0.04	-0.02	0.64^**^	0.48^**^	0.32^**^	0.11^**^	0.37^**^	0.27^**^	0.54^**^		
13	Perceived stigma	0.14^**^	0.12^**^	-0.05	-0.002	0.06	0.10^*^	0.07	-0.11^**^	0.20^**^	-0.02	0.10^*^	0.10^*^	
14	Desire for social distance	0.12^**^	0.14^**^	-0.02	-0.08^*^	0.32^**^	0.32^**^	0.19^**^	-0.13^**^	0.41^**^	0.05	0.29^**^	0.38^**^	0.49^**^

^a^ AUDs = Alcohol use disorders

^b^ It is treated as a nominal variable with multiple categories (biogenetic, psychosocial, biopsychosoical and other causal beliefs)

^c^ It is positively associated with biopsychosocial perspectives

^d^ A biogenetical perspective was associated with the lowest level of anger, followed by psychosocial and then biopsychosocial perspectives

We then examined the independent correlations of the desire for social distance using linear regression analysis. After controlling for age, gender, education, employment, lifetime mental health utilisation and alcohol consumption, a greater desire for social distance was independently associated with more recognition of AUDs as mental illnesses, increased perceived responsibility, a higher level of fear, less willingness to help, a greater tendency of avoidance, as well as increased perceived stigma (Table 4). Additionally, younger people and those who consumed more alcohol showed less desire for social distance. Together, all variables in the regression analysis, including the confounding variables, explained 40% of the variance in the desire for social distance from individuals with AUDs. All VIF values were less than 2.5, indicating low multicollinearity.

**Table 4:** Linear regression analysis on the desire for social distance, controlling for age, gender, education, employment, alcohol consumption and lifetime mental health service us (adjusted R^2^ = 0.41)

Independent variables	B^*^	SE^c*^	Beta^#^	*p*	95% CI^d^
AUD^a^ is a mental illness (0 = no, 1 = yes)	0.60	0.26	0.07	0.02	0.09–1.11
AUDs are due to a lack of willpower (0 = no, 1 = yes)	0.30	0.23	0.04	0.18	-0.14–0.74
Potential causes of AUDs^b^	-0.35	0.29	-0.04	0.23	-0.92–0.22
Supporting health insurance coverage (0 = yes, 1 = no)	0.11	0.22	0.01	0.60	0.54– -0.31
Dangerousness	0.12	0.07	0.06	0.09	-0.02–0.27
Responsibility	0.20	0.07	0.09	0.006	0.06–0.35
Treatability	0.05	0.06	0.03	0.44	-0.07– 0.17
Pity	-0.05	0.04	-0.04	0.22	-0.13–0.03
Anger	0.03	0.05	0.02	0.59	-0.07–0.13
Fear	0.20	0.06	0.13	0.002	0.08–0.32
Willingness to Help	-0.15	0.05	-0.09	0.004	-0.25–-0.05
Avoidance	0.27	0.05	0.18	< 0.001	0.18–0.36
Perceived stigma	0.34	0.03	0.37	< 0.001	0.29–0.39
Age	0.03	0.01	0.09	0.001	0.01– 0.05
Gender (0 = male, 1 = female)	-0.17	0.24	-0.02	0.49	-0.64–0.30
Education (years)	-0.08	0.09	0.03	0.38	0.11– -0.28
Employment (0 = no, 1 = yes)	-0.87	0.78	0.03	0.27	0.66– -2.39
Any mental health service uses, lifetime (0 = no, 1 = yes)	-0.18	0.29	-0.02	0.55	-0.75–0.40
Alcohol consumption	-0.22	0.05	-0.15	< 0.001	-0.31– -0.13

^a^ AUD = Alcohol use disorder. ^b^ reference category = biopsychosocial model. ^c^ SE = Standard Error

^d^ CI = Confidence Interval. * Unstandardised coefficient. ^#^ Standardised coefficient

**Fig. 1:**
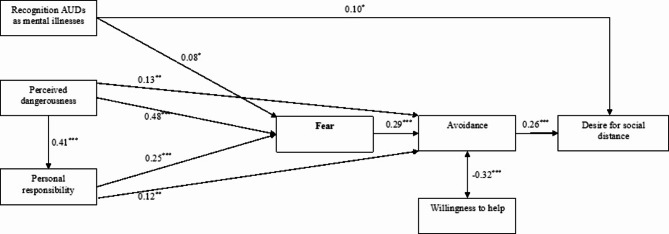
Path analysis of fear as a mediator between recognition of AUDs as mental illnesses, perceived dangerousness and personal responsibility and behavioural outcomes (avoidance, willingness to help and desire for social distance), controlling for age, gender, education, employment, alcohol consumption and lifetime mental health service use. Perceived stigma was not significantly associated with other variables and is therefore not shown in this model. Only significant standardised coeffients are displayed. ^*^p < 0.05, ^**^p < 0.01, ^***^p < 0.001

Based on the results of the regression analysis, we conducted a path analysis to test the mediation effect of fear on the relationships between the recognition of AUDs as mental illnesses, perceived stigma, perceived responsibility, and the desire for social distance. Perceived dangerousness, although marginally significant (*p* = 0.09) in its association with the desire for social distance, was also included in the path analysis due to its theoretical relevance within the attribution theory framework. The results showed that the recognition of AUDs as mental illnesses had both a direct and an indirect (ß = 0.006, *p* = 0.04) effect via fear on the desire for social distance, while fear fully mediated the effect of perceived dangerousness (ß = 0.15, *p* < 0.001) and responsibility (ß = 0.07, *p* < 0.001) on the desire for social distance (Fig. 1). Additionally, perceived dangerousness and responsibility influenced avoidance both directly and indirectly through fear (ß = 0.22, *p* < 0.001 and ß = 0.07, *p* < 0.001, respectively). Perceived stigma did not show any significant direct or indirect effects on the desire for social distance or avoidance. The model showed a good fit to the data (𝛘^2^/df = 1.8, *p* = 0.08; CFI = 0.99, TLI = 0.94, RMSEA = 0.04, SRMR = 0.02).

## Discussion

The present study provides initial evidence that a higher level of desire for social distance from individuals with AUDs is associated with greater recognition of AUD as mental illnesses, stronger perceptions of dangerousness and responsibility, higher levels of fear, a greater tendency to avoid, and less willingness to help among Chinese adults. Fear emerged as a pivotal factor, fully mediating the relationships between perceived dangerousness, personal responsibility, and social distance.

Contrary to previous findings [13], this study found that a higher level of recognition AUDs as mental illnesses was associated with increased desire for social distance from people with AUDs. In this study, the majority of participants labelled AUDs as mental illnesses and recognised the connection between AUDs and a biopsychosocial model. However, the recognitions coexist with perceptions of lack of willpower, dangerousness, perceived stigma, and a desire for social distance. It implies that alcohol-related stigma may be intertwined with the overall stigma surrounding mental illnesses. The results could help interpret previous findings that despite an increasing knowledge about alcohol use disorders, high levels of public stigma have persisted and, in some cases, worsened [23, 52]. While the recognition of alcohol use disorders as mental illnesses is essential for promoting a comprehensive approach to treatment and support [13, 53], equally critical is the need to address pervasive stigmatised attitudes associated with mental illness.

In China, treatment for AUDs is generally not covered by health insurance programs [54]. In case where some treatments are included, the extent of reimbursement for medical expense varies significantly based on specific insurance plans [55]. This lack of comprehensive coverage may exacerbate barriers to accessing care for individuals with AUDs. Remarkably, a significant majority of the respondents in this study opposed health insurance coverage for alcohol-related treatment, which was associated with perceptions of personal responsibility, unwillingness to help, avoidance and a desire for social distance. This stance could lead to discrimination within the healthcare system and further hinder the provision of adequate care [56].

In alignment with previous research [13, 23], heightened perceptions of dangerousness and responsibility were correlated with increased tendencies to avoid, elevated negative emotions such as pity, anger and fear, and a greater desire for social distance. Conversely, higher perceptions of dangerousness and treatment optimism were associated with a greater willingness to help. Safety concerns may drive individuals in collectivistic society, such as China, to engage in helping behaviours that mitigate perceived risks and benefit the collective community [57, 58]. Additionally, an optimistic attitude towards treatment could play a role, as individuals may believe that their assistance can contribute positively to the recovery journey of those with AUDs [59, 60].

However, the public perception of treatment optimism, a proxy of controllability [61], might also create an expectation that individuals should be able to manage their alcohol use problems with available treatments. The failure to meet expectations may result in increased blame and social rejection, especially in Chinese society that emphasise on self-control [6]. Recognising the dual nature of treatment optimism as a double-edged sword in addressing alcohol-related stigma, efforts should extend beyond merely promoting optimism about treatment outcomes to fostering a no-blame perception to reduce stigma and support recovery [62]. Given the significantly low treatment rate for AUDs in China [4], there is a high demand to promote treatment availability and accessibility of AUDs treatment.

Consistent with the reactions observed towards individuals with substance use disorders [6], the negative emotional responses, including pity, fear and anger, were associated with heightened perceived dangerousness, responsibilities and treatment optimism. Pity was associated with more willingness to help, which aligns with previous research indicating that pity might elicit compassion and thus promote supportive behaviour [12]. While pity is often considered as a moral virtue and reflects a compassionate response to someone’s suffering [63], it did not show a significant correlation with perceived stigma or a desire for social distance. When exploring the independent relationships between emotional reactions and the desire for social distance, only fear remained a significant association. The findings from the path analysis highlighted the crucial role of fear in driving social distancing behaviour toward individuals with AUDs, largely due to perceptions of danger or threat. This aligns with attribution theory [14, 15], which suggests that emotions like fear, elicited by stereotypes such as dangerousness and responsibility, can exacerbate discriminatory behaviours. These results emphasise the need for stigma-reduction interventions that focus on mitigating fear through public education, reframing narratives around dangerousness and responsibility, promoting understanding of AUDs as treatable conditions rather than as threats to personal safety, and fostering more inclusive attitudes towards individuals with AUDs.

While acknowledging AUDs as mental illnesses may increase awareness and potentially reduce stigma [13], it can also lead to heightened emotional reactions such as fear, which may counteract the benefits of greater recognition. In the Chinese cultural context, where collective harmony and the importance of maintaining “face” are highly valued [43], fear associated with mental illness labels may have unique implications. This underscores the complexity of addressing alcohol-related stigma in China, as well-intentioned public health messages may inadvertently trigger negative emotional responses that perpetuate social distancing behaviour. Contrary to expectations, while perceived stigma was significantly associated with the desire for social distance in the regression analysis, it showed neither direct nor indirect effects on the desire for social distance in the path analysis, suggesting its influence may be confounded by other variables such as fear, recognition of AUDs as mental illnesses, as well as perceived dangerousness and responsibility, which play a more prominent role in influencing the desire for social distance.

Some limitations to this study need to be considered. First, compared to the general Chinese population, this study had a higher proportion of female, younger participants with advanced education levels. Specifically, younger people in this study showed less desire for social distance towards individuals with AUDs, suggesting potentially fewer stigmatising attitudes compared to the general population. Future research with more representative samples is needed to validate the findings. Furthermore, the findings may not generalise well to those who are less active online or chose not to participate in this study. Second, no causality or potential direction of the associations can be drawn from this cross-sectional study. Future research employing a longitudinal design and mediation analysis could provide deeper insights into the mechanisms underlying stigma related to AUDs. Third, self-report desire for social distance, rather than observed discriminatory behaviour, was used as a proxy for measuring discrimination. Fourth, a few variables, including (mis-)conception and attributions, were assessed with a single item, which may limit the sensitivity and statistical power of the analyses. The use of multiple items could improve the reliability and validity of these constructs in future research. Fifth, the vignette methodology used in this study, while effective for standardising assessments, cannot fully capture the complex realities of AUDs or the diverse characteristics of individuals affected by the condition. Future research should include vignettes reflecting a wider range of profiles of AUDs to enhance ecological validity. Lastly, social desirability may lead to potential underreporting of stereotypes and the desire for social distance, while leading to an over-reporting of the willingness to help.

Despite these limitations, findings from this study have significant implications for stigma reduction and the improvement of social integration for individuals with AUDs. Reducing stigma requires a culturally nuanced approach that acknowledges the unique cultural heritage and social dynamics influencing perceptions of alcohol use in China. Cultural norms, such as its role in social bonding, contribute to perceptions of controllability and personal responsibility for AUDs. Therefore, public stigma reduction efforts should focus on reshaping cultural narratives and policies to promote understanding and empathy [30]. Highlighting the multifaceted causes of AUDs may help dispel misconceptions and stereotypes, fostering a more supportive environment for individuals with AUDs. Comprehensive public mental health programmes should address the broader stigma related to mental illnesses while also targeting the specific stigma associated with AUDs. Emphasising the biopsychosocial causes of AUDs, rather than framing them solely as mental illnesses, may help mitigate the risk of reinforcing stigma in cultural contexts where mental illness itself is highly stigmatised. Additionally, culturally sensitive anti-stigma campaigns should promote non-blaming causal attributions while addressing culturally ingrained attitudes and behaviours [64, 65]. Furthermore, interventions should foster inclusive environments through storytelling and contact-based strategies. Programmes like the Honest, Open, Proud peer-led group programme [66], which support individuals with lived experience to share their recovery stories, may enhance public respect and contribute to stigma change [8]. These efforts should be complemented by policy advocacy to address systemic stigma, such as workplace support programmes, insurance coverage for AUD treatment, and media guidelines that discourage stigmatising portrayals of AUDs. Addressing the significantly low treatment rate for AUDs in China is a critical component of these efforts, underscoring the urgent need to improve treatment availability and accessibility.

Future research should explore the cultural and policy-level factors shaping public perceptions and behaviours toward individuals with AUDs. Developing culturally tailored stigma reduction programmes for various stakeholders, including healthcare professionals, media outlets, policymakers, police officers, employers, and community networks, is essential to foster a supportive and inclusive environment for individuals with AUDs.

In conclusion, this study presents novel findings that recognition of AUDs as mental illnesses has a direct effect on the desire for social distance, while the perception of dangerousness and personal responsibility indirectly influence it, fully mediated by heightened fear. Culturally tailored approaches, such as contact-based strategies (e.g. sharing recovery stories), may help address perceptions of dangerousness and responsibility, reduce fear towards individuals with AUDs, and foster a more inclusive environment. These efforts could ultimately support the social integration and recovery of individuals with AUDs. Future research is needed to examine how these psychological processes interact with broader social and cultural contexts to guide the development of effective stigma-reduction strategies.

## Data Availability

The datasets used and analysed during the current study are available from the corresponding authors upon reasonable request.
